# Traditional Knowledge and Formulations of Medicinal Plants Used by the Traditional Medical Practitioners of Bangladesh to Treat Schizophrenia Like Psychosis

**DOI:** 10.1155/2014/679810

**Published:** 2014-06-30

**Authors:** Md. Nasir Ahmed, Md. Nur Kabidul Azam

**Affiliations:** Ethnobotany & Ethnomedicine Division, TechB Herbal Solution, Bheramara, Kushtia 7040, Bangladesh

## Abstract

Schizophrenia is a subtle disorder of brain development and plasticity; it affects the most basic human processes of perception, emotion, and judgment. In Bangladesh the traditional medical practitioners of rural and remote areas characterized the schizophrenia as an insanity or a mental problem due to possession by ghosts or evil spirits and they have used various plant species' to treat such symptoms. The aim of the present study was to conduct an ethnomedicinal plant survey and documentation of the formulations of different plant parts used by the traditional medical practitioners of Rangamati district of Bangladesh for the treatment of schizophrenia like psychosis. It was observed that the traditional medical practitioners used a total of 15 plant species to make 14 formulations. The plants were divided into 13 families, used for treatment of schizophrenia and accompanying symptoms like hallucination, depression, oversleeping or insomnia, deterioration of personal hygiene, forgetfulness, and fear due to evil spirits like genies or ghost. A search of the relevant scientific literatures showed that a number of plants used by the medicinal practitioners have been scientifically validated in their uses and traditional medicinal knowledge has been a means towards the discovery of many modern medicines. Moreover, the antipsychotic drug reserpine, isolated from the dried root of *Rauvolfia serpentina* species, revolutionized the treatment of schizophrenia. So it is very much possible that formulations of the practitioner, when examined scientifically in their entireties, can form discovery of lead compounds which can be used as safe and effective antipsychotic drug to treat schizophrenia.

## 1. Introduction

Schizophrenia is a psychological disorder characterized by psychotic symptoms: hallucinations and delusions that significantly affect emotions, behavior, and, most notably, mental processes and mental contents. Psychosis is a condition of mental illness that causes a person to lose his or her sense of reality. Schizophrenia is a type of psychosis meaning spilt mind which often describes as a mental disorder characterized by impairments in the perception or expression of reality and by significant social or occupational dysfunction. People with this disease have delusions, hallucinations, disorganized speech, grossly disorganized behavior, or catatonic behavior [1, 2]. The world over, from China or Finland to the United States or New Guinea, approximately 1% of the population will develop schizophrenia at some point in their lives [3, 4]. Schizophrenia is one of the top five causes of disability among adults in developed nations, ranking with heart disease, arthritis, drug use, and HIV [5]. In the United States, about 5% of people with schizophrenia (about 100,000 individuals) are homeless, 5% are in hospitals, and 6% are in jail or prison [6]. Together, these three groups of people represent about 16% of Americans with schizophrenia; in contrast, 34% of people with this disorder live independently. According to World Health Organization (WHO) at least 40 million people in the world suffer from mental disorders such as schizophrenia and dementia [7]. Over 10% of schizophrenia patients ultimately commit suicide [8, 9]; this makes the disease a serious psychiatric illness among the various nationalities of the world.

Bangladesh is a densely populated area where prevalence of psychiatric illness is not less than that of any other country in the world. A study showed that 29% of patients attending general practice were suffering from functional disorder and 6% from both functional and organic disorders. The same study demonstrated that 47% of patients were suffering from neurotic disorder, 37% from psychosomatic disorder, 10% from affective disorder, 1.44% from schizophrenia, 2.88% from substance use disorder, and 2% from organic psychiatric syndrome [10]. Another study conducted in Outpatient Department of National Institute of Mental Health (NIMH), Dhaka, Bangladesh, revealed that 37.4% of patients were suffering from schizophrenia and schizophrenia like psychotic disorders, 16.14% from anxiety disorders, 11.19% from major depressive disorder, and 8.95% from bipolar mood disorder [11]. In the Dhaka city, most of the patients (70.4%) were from urban background and from rural area 29.6% patients [12]. According to other studies it was seen that across various countries, schizophrenia is more common among people in urban areas and lower socioeconomic classes than among people in rural areas and higher socioeconomic classes [13, 14].

Antipsychotic agents are the cornerstone of acute and maintenance treatment of schizophrenia and are effective in the treatment of hallucinations, delusions, and thought disorders, regardless of etiology. In recent decades, the modern psychiatry has introduced herbal medicine in the treatment of psychiatric disorders including schizophrenia. After the rise of a pharmaceutical industry in the last century and significant progress in the treatment, a period of disappointment comes in accepting the fact that synthetic drugs are not almighty. Due to this fact, there has been a growing interest in the last decades for the treatment of psychiatric disorders including schizophrenia by using alternative and complementary methods [15] and a study revealed that 44% of psychiatric patients with schizophrenia (9%) had used herbal medicine (mainly for psychiatric purposes) during the previous 12 months [16].

Plants contain various phytochemicals and these phytochemicals can play an important role in reducing occurrences of many diseases by boosting up various organ functions of the human body. Many traditional healing herbs and their parts have been shown to have medicinal value and can be used to prevent, alleviate, or cure several human diseases [17]. It has also been observed that number of modern drugs has been derived from plants used by the indigenous people [18]. Modern drugs like aspirin, atropine, ephedrine, digoxin, morphine, quinine, reserpine, and tubocurarine are examples, which were originally discovered through observations of traditional cure methods of indigenous peoples [19]. Presently, there is a resurgence of herbal medicine as people want more control in their personal healthcare. The U.S. herbal market is growing tremendously with consumer demand way ahead of regulatory agencies. It is interesting to note that four (Ginkgo, St. John's Wort, Valerian, and Kava) of the top ten herbs purchased in the U.S. (according to 1999 Whole Foods Survey) have psychotropic activity. Recent trends in research into African plant uses show that traditional medicine is commonly used to treat neurological disorders in the West African region, and some recent publications are available [20].

In Bangladesh, traditional medical practice is still ongoing. The traditional medical practitioners (generally known as* Kabirajes* by the mainstream community) perform a central role in providing primary healthcare to the rural inhabitants of Bangladesh. There are 86,000 villages in the country and almost every village has one or two traditional* Kabirajes*. They are the providers of primary healthcare to village populations of Bangladesh and moreover they are the important sources of ethnomedicinal knowledge. The practices of the* Kabirajes* extend throughout both urban and rural areas of the country, although rural practice is more extensive than urban practice.

The advantage of the* Kabirajes* is that they rely chiefly on medicinal plants for treatment. The formulations are rarely complex and usually consist of simple extraction of juice of whole plant or plant part through squeezing plant part. The juice is then administered orally or typically applied depending on the nature of diseases. The previous ethnomedico-botanical studies conducted among folk and tribal medicinal practitioners of the country have noticed considerable variation between the medicinal plants selected by different* Kabirajes* for treatment of a given ailment [21–25]. The aim of the present study was to document the traditional medicine knowledge as well formulations of medicinal plants used by the traditional medical practitioners of Rangamati district in the Chittagong Hill Tracts (CHT) region of Bangladesh for the treatment of schizophrenia like psychosis.

## 2. Materials and Methods

### 2.1. Study Area

**Figure 1:**
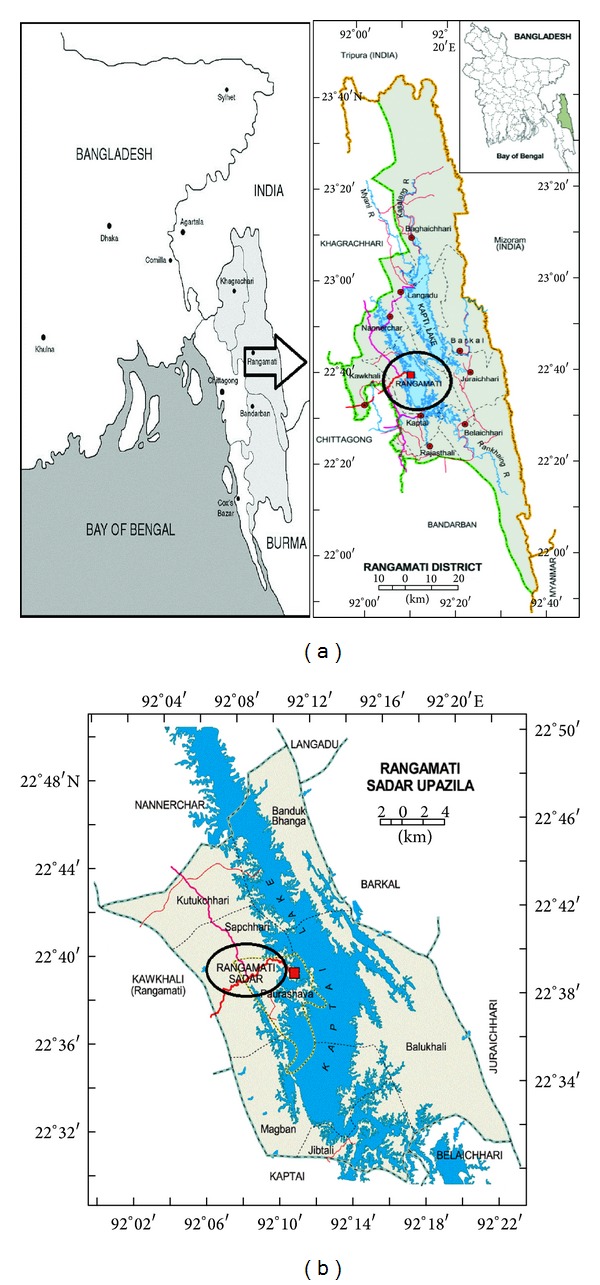
Map of Bangladesh showing the Rangamati district. Study site is marked on the map with black round shade.

For the present study, survey was done between March to Mid-April' 2013, in the largest district of the country called Rangamati which is located in the CHT region. It is consists of 10 upazilas and bordered by the Tripura state of India to the north and Bandarban district to the south. The area of the district is 6116 km^2^ of which 1292 km^2^ is riverine and 4825 km^2^ is under forest vegetation. The main survey was conducted in the Rangamati Sadar Upazila (located at 22.6500°N 92.1833°E), which consists of 6 unions, on the information about medicinal plants commonly used for treatment of psychotic disorders with particular attention to schizophrenia. The study area for this specific ethnomedicinal survey was selected by the authors themselves regarding information on the noted traditional medical practices. Moreover, the study area as well as the total CHT region is very rich in species diversity. We are conducting systematic ethnomedicinal survey of Bangladesh since 2011 as to explore how do the* Kabirajes* of Bangladesh select medicinal plants for treatment of any ailment. In order to address such question we conducted this specific survey among* Kabirajes* of Rangamati Sadar Upazila of Bangladesh (Figure 1). The above question is more important from the viewpoint that how and why medicinal plants are selected by the TMPs can lead the way towards a better linking between traditional knowledge with biomedical science [26, 27].

### 2.2. Mode of Interview and Plant Data Collection

The collection of data through interviews of 3 traditional medical practitioners (TMP; aged between 61–67) was conducted by the researchers themselves with the help of a semistructured questionnaire. The number of 3 TMPs was chosen on the basis of recommendation of the local inhabitants as to being more proficient and reputed to their practices. In the preliminary survey, the TMPs were specifically asked as to whether they know and treat schizophrenia, the basis for their diagnosis, and their mode of treatment when the disease has been diagnosed as schizophrenia. According to folk medical practitioners, the symptoms were hallucinations, over sleeping in the day, insomnia, staying alone in the dark room, anxiety, being silent or not so much interested to keep attached with family members, and most commonly getting fear due to invisible person (as per patient's thoughts). The traditional medical practitioners defined this fear to be due to evil spirits, genies, or ghosts. The basic method followed was one of guided fieldwork [28–30]. Information collected from the* Kabirajes* consisted of medicinal plants or plant parts used, formulations, and dosage. The information was noted down during the daytime interviews. At the end of each interview plant specimens shown by the traditional medical practitioners were collected, dried, and later brought back to the Dhaka for identification at the Bangladesh National Herbarium and all the voucher specimens were deposited there. Nomenclature of plants was compiled from the plant list database [31]. For this specific ethnomedicinal survey, the number of visits was made to the traditional medical practitioners to gain their confidence.

## 3. Results

**Figure 2:**
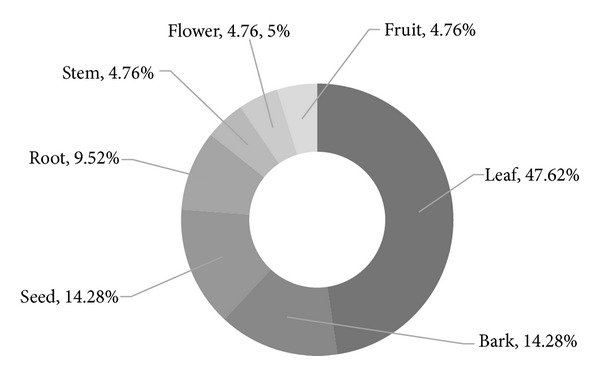
Parts of medicinal plants used by the traditional medical practitioners of Rangamati Sadar Upazila to treat schizophrenia like psychosis.

It was observed that the 3 traditional medical practitioners (TMP) of the Rangamati district used a total of 14 formulations from the 15 plant species, distributed into 13 families for treatment of schizophrenia like psychotic episodes. The Rubiaceae and Lamiaceae families were contributing 2 plants, the rest of the plant families contributed one plant each. The various plant parts were used including leaf, roots, bark, stem, flower, seed, and fruit. This study recorded that several parts or individual plant species are used as medicine. Leaves constituted the major plant part used, forming 47.62% of total uses. Barks and seeds each constituted 14.28% of total uses. The other plant parts mentioned constituted, respectively, 9.52, 4.76, 4.76, and 4.76% of total uses (Figure 2).

The medicinal knowledge of TMPs was derived from the earlier generation practitioners, with whom every TMP had to serve a period of apprenticeship and training before they could practice independently. This medicinal knowledge has come out of practice through the centuries and every practitioner had their own lists of plants for treatment of any particular disease or disorder. As mentioned earlier all 3 TMPs were interviewed separately, so the plants advised by the TMPs did not reflect any individual opinion. It can be concluded by the interviewers that schizophrenia and other related psychotic problems have existed for centuries among the people surveyed and various TMPs have developed their own medicinal plant formulations for treatment of schizophrenia like psychosis, possibly through trial and error methods over centuries long practices.

Any sort of complex formulations was avoided by the TMPs for the treatment of schizophrenia like psychosis. Generally fruits and seeds were consumed directly or with water. In most cases, juice obtained from squeezed plant part was advised to be taken or pills made from paste of plant part taken orally. It was noted that mashed leaves of* Piper retrofractum* are taken with rice. A combination of bark and seed of* Thevetia peruviana* paste is taken orally. Another formulation suggested by a TMP was that juice from macerated* Euphorbia neriifolia* leaves is taken orally; the formulation was also used to keep head cool and to increase memory of a patient as per the TMP. There were also mentioned some more easy formulations: smell of flower of* Abroma augusta* is taken with the nose and leaves of* Vitex negundo* were carried alongside the body. If a patient is in severe condition then the leaves of* Cannabis sativa* were used to make vapor and the vapor is taken by the nose, as advised by a TMP. Leaf juice of* Ocimum americanum* is mixed with seeds of* Brassica juncea* and taken orally till cure. Most of the formulations were usually administrated orally with three exceptions. The rest of the formulations were given in Table 1.

In our present study, TMPs recommended different forms of medication including juice (3 species), pill, vegetable and paste (2 each), tablet, vapor, oil message, decoction (1 species each), and others (3). Most often standard medicines are prescribed in mixed ingredient form by mixing several valuable medicinal plants or several plant parts of one plant species. TMPs believe that using plant or plant part mixtures in the preparation of medicine is important as a single plant alone may not be sufficient to cure any disease completely and the combination of several medicinal plants increases the quality and efficacy of medicine. Similar observations have also been found in Nepal and India [32, 33].

## 4. Discussions

Investigation into the mechanism of action of the drugs used to treat schizophrenia has not provided clear understanding of the pathogenesis of the disease. While schizophrenia is highly heritable, the genetics are complex and the interpretation of genetic data has proven difficult. Now advances in phenotypic analysis, neuroimaging, genetics, and molecular pathology provide the basis for optimism [34]. Schizophrenia can be understood as a subtle disorder of brain development [35–37].

### 4.1. Neural Communication in Schizophrenia

Recent findings have shown changes in markers in a number of neurotransmitter systems in the brains of subjects with schizophrenia which include the dopaminergic, serotonergic, glutamatergic, and GABAergic systems of the CNS. Many of these changes also appear to be regionally specific, and abnormalities in nonneurotransmitter specific pathways have been found in schizophrenia [38]. A major component of the studies used postmortem CNS tissue because these sites are amenable to manipulation by therapeutic agents, in many cases they are the sites of action of drugs which provide antipsychotic activity [39]. Ultimately neurobiology of schizophrenia may help illuminate the nature of normal thought, perception and emotion.

The findings that antipsychotic drugs are dopamine D2 receptor antagonists and the* dopamine hypothesis* propose that an overproduction of dopamine or an increase in the number or sensitivity of dopamine receptors is responsible for schizophrenia. According to this hypothesis, the excess dopamine or extra sensitivity to this neurotransmitter triggers a flood of unrelated thoughts, feelings, and perceptions. Delusions are then attempt to organize these disconnected events into a coherent, understandable experience [40, 41].

There has been an increasing acceptance that antipsychotic drugs that bind to both the dopamine D_2_-like receptor family and the* serotonin *(5HT)_2A_ receptor have been improved clinical outcomes [42]. Using DNA from peripheral tissue, a number of studies have suggested that mutations in the gene for the 5HT_2A_ receptor are associated with schizophrenia [43].

The hypothesis that changed* glutamatergic* function is involved in the pathology of schizophrenia and a glutamate receptor ion channel blocker reduce or exacerbate a schizophrenia like psychosis [44]. The N-methyl-D-aspartate (NMDA) glutamate receptor has been shown to play a crucial role in learning and memory, and hence abnormalities in its functioning may explain some of the deficits associated with schizophrenia, such as deficits in working memory. Studies have found unusually high levels of glutamate in people with schizophrenia, particularly in the frontal lobe [45, 46]; such an excess of glutamate may disrupt the timing of neural activation in the frontal lobe, which in turn may impair cognitive activities that depend on the smooth coordination of different operations, such as working memory, where information must be actively retained in short-term memory as it is operated upon [47].

Research findings suggest that stress can contribute to schizophrenia because stress affects* cortisol* production, which in turn affects the brain. The relationship with cortisol appears even in childhood. Children who are at risk for schizophrenia react more strongly to stress, and their baseline levels of cortisol are higher than those of other children [48]. The relationship between stress, cortisol, and symptoms of schizophrenia has also been noted during adolescence [49]. Even after adolescence, people with schizophrenia have higher levels of stress-related hormones, including cortisol [50].

Several lines of evidence implicated the* GABAergic* system in the pathology of schizophrenia. The study of mRNA encoding the different subunits has now extended original findings on radioligand binding to show an increase in levels of mRNA encoding the *α*-1 subunit of the GABA_A_ receptor in Brodmann's areas 9 and 10 from subjects with schizophrenia [51]. There are changes in expression of GABA_A_ receptor subunit in schizophrenia which are the finding that there is a marked decrease in levels of mRNA encoding for the short form of the *γ*2 subunit of the GABA_A_ receptor in the prefrontal cortex from schizophrenia [52].

### 4.2. Herbal Medications in the Enhancement of Brain Function

Humans consume a wide range of foods, drugs, and dietary supplements that are derived from plants and which modify the functioning of the central nervous system (CNS). The psychoactive properties of these substances are attributable to the presence of plant secondary metabolites; in many cases, the effects of these phytochemicals on the human CNS might be linked either to their ecological roles in the life of the plant or to molecular and biochemical similarities in the biology of plants and higher animals [53]. Schizophrenia as well as other psychotic disorders is likely to involve a complex interplay of many brain systems and neurotransmitters including dopamine, serotonin, and glutamate, and there is extensive evidence that schizophrenia is a biological disease of the brain. The primary mechanism of action involves modulation of neuronal communication, via specific plant metabolites binding to neurotransmitter/neuromodulator receptors [54] and via alteration of neurotransmitter synthesis and general function [55]. Other mechanisms involve stimulating or sedating CNS activity and regulating or supporting the healthy function of endocrine system [56, 57]. Traditional antipsychotics have been the first step in treating schizophrenia, that is, thorazine (chlorpromazine) is dopamine antagonist, which effectively blocks the action of dopamine and diminishes positive symptoms in approximately 75–80% of people with schizophrenia who take such antipsychotic medication [58]. Traditional antipsychotics have sedative properties which affect patients quickly; such sedation and improvement in psychotic symptoms can take anywhere from 5 days to 6 weeks [59].

A huge scientific literature focusing on psychoactive herbal extracts and their phytochemicals, encompassing hundreds of thousands of scientific papers, has emerged over recent decades. The vast majority of these papers describe* in vitro* investigations of the potential mechanisms of action of putatively psychoactive phytochemicals, whereas a much smaller proportion explores their effects* in vivo* on animals and only a tiny minority investigates their efficacy in humans. Zhang [60] identified extracts and constituents from 85 individual medicinal plants that have demonstrated potential efficacy for treating psychiatric disorders on the basis of animal behavioral models alone.

A study indicated antipsychotic activity along with antianxiety and anticonvulsant from the hydroalcoholic extract of* Euphorbia neriifolia* leaves in mice and rats [61]. The observation of another study indicated that the anticataleptic activity exhibited by ethanolic extract of* Randia dumetorum* Lam fruits does not possess antidopaminergic and antiserotonergic activity. Catalepsy usually associated with catatonic schizophrenia [62].

Methanolic extract of* Aegle marmelos* leaves possessed potential anxiolytic and antidepressant activities in albino mice and also may be served as a potential resource for natural psychotherapeutic agent [63]. In India,* Aegle marmelos* highly reputed ayurvedic medicinal tree and has been used in nervous disorder and as tonic for brain [64, 65]. A survey study conducted by Rahmatullah et al. [66, 67] reported that TMP of Santals tribe in Bangladesh used* Coccinia grandis* for the treatment of mental disorder.

*Ocimum americanum* possessed relaxant effects [68] and can be considered as beneficial to patients with mental disorders. The seed of* Brassica juncea* and leaves juice of* Ocimum americanum* were mixed with leaves of* Acorus calamus* L. (Acoraceae) for treating mental disorders as well being possessed with evil spirits; such combined formulation was used by the TMP of the Tonchongya tribe of Bangladesh, and whether such beings exist or not is scientifically debatable, any possession by “evil spirits” can be considered as a sort of mental disorder with the status of the patient being in a state of delirium [69].* A. calamus* is also used to make brahmyadiyoga (an Ayurvedic medicine system) and has been used for the treatment of schizophrenia. An important general issue is the cost of treatment, as Ayurvedic treatments are cheaper and therefore more accessible to poor people than chlorpromazine, let alone the more recent atypical antipsychotics [70, 71].

The effect of acetone soluble fraction of methanolic extract of roots of the* Vitex negundo* (50 and 100 mg/kg, i.p.) was studied on haloperidol-induced catalepsy in mice, amphetamine induced stereotyped behaviour in rats, and dopamine-induced contraction isolated vas deferens preparation of rat. The acetone soluble fraction of methanolic extract of isolated of* V. negundo* significantly potentiated haloperidol induced catalepsy, antagonized dose dependent amphetamine-induced stereotyped behaviour, and also antagonized dopamine induced contractions of rat vas deference. The result suggests that the methanolic extract of* Vitex negundo* possessed antidopaminergic principle [72]. Another study results in mice indicated that roots of* Vitex negundo* have an effective anxiolytic agent [73].

Since the discovery of the endocannabinoid system, a growing body of psychiatric research has emerged focusing on the role of this system in major psychiatric disorders like schizophrenia, bipolar disorder, major depression, and anxiety disorder. A compound found in* Cannabis sativa* can treat schizophrenia as effectively as antipsychotic medications, with far fewer side effects, according to a preliminary clinical trial. Researchers led by Markus Leweke of the University of Cologne in Germany studied 39 people with schizophrenia who were hospitalized for a psychotic episode. Nineteen patients were treated with amisulpride, an antipsychotic medication that is not approved in the U.S. but is comparable to other medications that are antipsychotic. The rest of the patients were given cannabidiol (CBD), a substance found in* C. sativa* that is thought to be responsible for some of its mellowing or anxiety-reducing effects [74]. Unlike the main ingredient in marijuana, THC, which can produce psychotic reactions and may worsen schizophrenia, CBD has antipsychotic effects, according to previous research in both animals and humans. The use of CBD for schizophrenia is becoming more and more common. Studies from around the world are showing great promise [75]. Moreover, fMRI results strongly suggest that the antipsychotic effects of CBD involve the striatum and temporal cortex that have been traditionally associated with psychosis [76].

Antipsychotic-like activity of* Morinda citrifolia* in mice demonstrated the antidopaminergic effect, suggesting that this plant has antipsychotic-like activity which can be utilized in the treatment of psychiatric disorders [77].

No available scientific literature is found regarding antipsychotic effects of six plants which were used by the traditional medical practitioners' of Rangamati Sadar Upazila, Bangladesh, and these six species are* A. augusta* L.f,* L. polyantha* Juss.,* P. retrofractum* Vahl.,* F. hirta* Vahl.,* T. peruviana* (Pers.) K. Schum, and* Datura metel* L. However, it is very much important to conduct proper scientific study for the evaluation of antipsychotic effects of these plants. Bangladesh has a very rich diversity of medicinal plant species which has been used by the traditional healers of several districts for the treatment of different diseases like mental health problems, brain disorders, malaria, cardiovascular disorders, diabetes, tumor, snake bite, and rheumatoid arthritis [78–85]. Thus the body of the existing ethnomedical knowledge has led to great developments in schizophrenia as well as can be the source for the discovery of new antipsychotic drugs.

## 5. Conclusions

The use of psychoactive plants is practically a universal human phenomenon. Except for a few cultures lacking access to psychoactive plants, humans tend to use them routinely. The psychological and behavioral effects of such plants have been long recognized and information about their uses has been passed down through generations. However, with the advent of modern science we are able to better understand the composition of these plants and how they interact with the nervous system.

The available scientific literature strongly suggests that at least some of the plants used by the traditional medical practitioners of Bangladesh may have strong scientific basis for their use in the treatment of schizophrenia like psychotic episodes. This ethnobotanical data provides an interesting basis for further phytotherapeutical research towards discovery of novel and efficacious antipsychotic drugs, especially concerning uncommonly used species (i.e.,* A. augusta* L.f,* L. polyantha* Juss.,* P. retrofractum* Vahl.,* F. hirta* Vahl.,* T. peruviana* (Pers.) K. Schum, and* Datura metel* L.). Although some formulations have drawn attention, in depth clinical trials of each one of the plants presented in the table should be conducted which will be a major tool to prove the benefits for a schizophrenia patient as well as to evaluate more scientific researches for discovering the new antipsychotic drugs with giving less side effects.

## Figures and Tables

**Table 1:** Medicinal plants used by the traditional medical practitioners of Rangamati district for treatment of schizophrenia like psychotic episodes.

Serial number	Formulations and dosage	Parts used	Botanical name/vernacular name
1	Leaves of *Piper retrofractum *(Piperaceae) are taken orally in mashed form as vegetable with rice till cure.	Leaf	*Piper retrofractum *Vahl/Choi

2	Juice obtained from macerated leaves or roots of *Ficus hirta *(Moraceae) is taken orally till cure.	Leaf, root	*Ficus hirta *Vahl./Pakur

3	A combination of bark and seed of *Thevetia peruviana *(Apocynaceae) paste is taken orally till cure.	Bark, seed	*Thevetia peruviana *(Pers.) K. Schum./Holde Korobi

4	A combination of leaf and seed paste from *Datura metel *L. (Solanaceae) is taken orally or macerated leaves are pressed to the nose.	Leaf, seed	*Datura metel *L./Dhutura

5	Juice from macerated *Euphorbia neriifolia *(Euphorbiaceae) leaves is taken orally.	Leaf	*Euphorbia neriifolia *L./Monshaseez

6	Juice is collected from macerated bark of *Randia dumetorum *(Rubiaceae). It is then dried and turned into powder, which is mixed with sugar. Tablets weighing 5 g each are made from the mixture and taken till cure	Bark	*Randia dumetorum *(Retz.) Lam/Monkata

7	*Aegle marmelos *(Rutaceae) leaves and roots crushed and made into 1/2 tola amount pills. Pills are taken twice daily for three days. (1 tola = 11.66 g)	Leaf, root	*Aegle marmelos *(L.) Corr./Bel

8	*Litsea polyantha* (Lauraceae) bark is macerated with 1 g sugar to obtain juice, which is then taken orally.	Bark	*Litsea polyantha * Juss./Uruijja, Menda

9	Leaves of *Coccinia grandis* (Cucurbitaceae) are boiled and the decoction is taken orally.	Leaf	*Coccinia grandis* (L.) J. Voigt/Telakuchi

10	Leaf juice of *Ocimum americanum* (Lamiaceae) are mixed with mashed seeds of *Brassica juncea* (Cruciferae) and taken orally till cure.	Leaf, seed	*Ocimum americanum *L./Radha tulshi *Brassica juncea *(L.) Czern./Shorisha

11	Smell of flower of* Abroma augusta *(Malvaceae) is taken with the nose or crushed stems are taken with *Aloe vera* and sugar twice daily for seven days till cure.	Flower, steam	*Abroma augusta *L.f./Ulot Kombol

12	Leaves of *Vitex negundo* (Lamiaceae) are kept or carried alongside the body. Another formulation is to take pills in the morning made from the leaves.	Leaf	*Vitex negundo *L./Nishinda

13	Leaves from *Cannabis Sativa *(Cannabaceae) are used to make oil then message on the scalp till cure. If a patient is in severe condition then the leaves were used to make vapor and the vapor is taken by the nose.	Leaf	*Cannabis Sativa *L./Bhang, Siddhi

14	Ripe fruit of *Morinda citrifolia *(Rubiaceae) is eaten as raw and mashed leaves were eaten as vegetable.	Fruit, leaf	*Morinda citrifolia* Linn./Holdi Kachu, Noni
